# Whole Lung Irradiation after High-Dose Busulfan/Melphalan in Ewing Sarcoma with Lung Metastases: An Italian Sarcoma Group and Associazione Italiana Ematologia Oncologia Pediatrica Joint Study

**DOI:** 10.3390/cancers13112789

**Published:** 2021-06-03

**Authors:** Massimo E. Abate, Silvia Cammelli, Letizia Ronchi, Barbara Diletto, Lorenza Gandola, Anna Paioli, Alessandra Longhi, Emanuela Palmerini, Nadia Puma, Angela Tamburini, Maurizio Mascarin, Elisa Coassin, Arcangelo Prete, Sebastian D. Asaftei, Carla Manzitti, Gianni Bisogno, Marta Pierobon, Luca Coccoli, Mariella Capasso, Giovanni Grignani, Giuseppe M. Milano, Valentina Kiren, Franca Fagioli, Stefano Ferrari, Piero Picci, Elisa Carretta, Roberto Luksch

**Affiliations:** 1 Paediatric Oncology, AORN Santobono-Pausilipon, 80123 Napoli, Italy; m.capasso@santobonopausilipon.it; 2 Radiation Oncology, IRCCS Azienda Ospedaliero-Universitaria di Bologna, 40138 Bologna, Italy; silvia.cammelli2@unibo.it (S.C.); or letironchi@gmail.com (L.R.); 3 Department of Experimental, Diagnostic and Specialty Medicine-DIMES-Alma Mater Studiorum Bologna University, 40126 Bologna, Italy; 4 Radiotherapy, Fondazione IRCCS Istituto Nazionale dei Tumori, 20133 Milano, Italy; barbara.diletto@istitutotumori.mi.it (B.D.); lorenza.gandola@istitutotumori.mi.it (L.G.); 5 Medical Oncology, IRCCS Istituto Ortopedico Rizzoli, 40136 Bologna, Italy; anna.paioli@ior.it (A.P.); alessandra.longhi@ior.it (A.L.); emanuela.palmerini@ior.it (E.P.); 6 Paediatric Oncology, Fondazione IRCCS Istituto Nazionale dei Tumori, 20133 Milano, Italy; Nadia.Puma@istitutotumori.mi.it (N.P.); roberto.luksch@istitutotumori.mi.it (R.L.); 7 Haematology Oncology, Children’s University Hospital A. Meyer, 50139 Firenze, Italy; a.tamburini@meyer.it; 8 AYA Oncology and Pediatric Radiotherapy Unit, Centro di Riferimento Oncologico CRO IRCCS, 33081 Aviano (PN), Italy; mascarin@cro.it (M.M.); ecoassin@cro.it (E.C.); 9 Paediatric Haematology Oncology, IRCCS Azienda Ospedaliero-Universitaria di Bologna, 40138 Bologna, Italy; arcangelo.prete@aosp.bo.it; 10 Paediatric Haematology Oncology, OIRM Sant’ANNA, 10126 Torino, Italy; sebastiandorin.asaftei@unito.it (S.D.A.); franca.fagioli@unito.it (F.F.); 11 Paediatric Oncology, Gaslini Hospital, 16147 Genova, Italy; carlamanzitti@ospedale-gaslini.ge.it; 12 Paediatric Haematology Oncology, Azienda Ospedaliera di Padova, 35121 Padova, Italy; gianni.bisogno@unipd.it (G.B.); marta.pierobon@aopd.veneto.it (M.P.); 13 Paediatric Haematology Oncology, S. Chiara University Hospital, 56126 Pisa, Italy; l.coccoli@ao-pisa.toscana.it; 14 Division of Medical Oncology, Candiolo Cancer Institute, FPO-IRCCS, Strada Provinciale 142, Candiolo, 10060 Torino, Italy; giovanni.grignani@ircc.it; 15 Paediatric Haematology and Oncology, IRCCS Bambino Gesù Children’s Hospital, 00165 Roma, Italy; giuseppemaria.milano@opbg.net; 16 Paediatric Haematology Oncology, IRCCS Burlo Garofolo, 34137 Trieste, Italy; valentina.kiren@burlo.trieste.it; 17 I.S.G. Italian Sarcoma Group, 40136 Bologna, Italy; stefanoferrari.19855@gmail.com (S.F.); piero.picci@italiansarcomagroup.org (P.P.); 18 PhD Statistician, IRCCS Istituto Ortopedico Rizzoli, 40136 Bologna, Italy; elisa.carretta@ior.it

**Keywords:** Ewing sarcoma, pulmonary metastasis, busulfan, melphalan, lung irradiation, oncology

## Abstract

**Simple Summary:**

The lung is the most frequent site of metastasis in Ewing sarcoma, the second most common bone cancer affecting children, adolescents and young adults. The five-year overall survival of patients with isolated lung metastasis is approximately 50% after multimodal treatments including chemotherapy, surgery and radiotherapy. This retrospective study aimed to investigate the feasibility and the predictors of survival in 68 Ewing sarcoma patients with lung metastases who received high-dose chemotherapy with busulfan and melphalan, followed by reduced dose whole-lung irradiation, as part of two prospective and consecutive treatment protocols. This combined treatment strategy is feasible and might contribute to the disease control in lung metastatic Ewing sarcoma with responsive disease. Furthermore, the results of this study provide support to explore the treatment stratification for lung metastatic Ewing sarcoma based on the histological response of the primary tumor.

**Abstract:**

Purpose: To analyze toxicity and outcome predictors in Ewing sarcoma patients with lung metastases treated with busulfan and melphalan (BU-MEL) followed by whole-lung irradiation (WLI). Methods: This retrospective study included 68 lung metastatic Ewing Sarcoma patients who underwent WLI after BU-MEL with autologous stem cell transplantation, as part of two prospective and consecutive treatment protocols. WLI 12 Gy for <14 years old and 15 Gy for ≥14 years old patients were applied at least eight weeks after BU-MEL. Toxicity, overall survival (OS), event-free survival (EFS) and pulmonary relapse-free survival (PRFS) were estimated and analyzed. Results: After WLI, grade 1–2 and grade 3 clinical toxicity was reported in 16.2% and 5.9% patients, respectively. The five-year OS, EFS and PRFS with 95% confidence interval (CI) were 69.8% (57.1–79.3), 61.2% (48.4–71.7) and 70.5% (56.3–80.8), respectively. Patients with good histological necrosis of the primary tumor after neoadjuvant chemotherapy showed a significant decreased risk of pulmonary relapse or death compared to patients with poor histological necrosis. Conclusions: WLI at recommended doses and time interval after BU-MEL is feasible and might contribute to the disease control in Ewing sarcoma with lung metastases and responsive disease. Further studies are needed to explore the treatment stratification based on the histological response of the primary tumor.

## 1. Introduction

Ewing sarcoma (ES) is the second most common bone tumor affecting children, adolescents and young adults [1]. Approximately 25% of patients with ES have metastatic disease at diagnosis and the lung is the most frequent site of metastasis [2,3]. In the last 30 years only slight improvements in overall survival (OS) and event-free survival (EFS) were achieved even with multimodal approaches [4,5,6,7,8,9]. Although the current five-year OS for patients with localized disease is 65% to 75% [7,8,9,10], patients with metastases have a long-term survival less than 35% [3,11], except for those with isolated lung metastasis in whom the five-year OS is approximately 50% [12,13,14]. The relapsed disease still represents a challenge due to a dismal prognosis [15,16,17].

Previous multicentric trials showed a therapeutic benefit for WLI in ES patients with lung metastases [3,18,19]. In international guidelines WLI is strongly recommended for metastatic disease to the lungs at doses of 15 or 18 Gray (Gy), according to the age of the patient [20]. Other studies suggested that the impact on the outcome of the different treatment modalities, including WLI, needs to be better defined [21,22,23].

In the most recent European trials [7,10,12,13,14], BU-MEL has been used to consolidate treatment in high-risk ES. In the EuroEwing 99 and Ewing 2008 trials, the superiority of high-dose chemotherapy (HDCT) with BU-MEL, over standard chemotherapy, resulted statistically significant in high-risk localized disease [10].

With the intent to intensify the multimodal treatment in lung metastatic ES patients, HDCT combined with WLI was performed in different clinical trials [3,13,18,19,24]. However, the treatment heterogeneity and the different patient cohorts shown across the studies make the role of HDCT and WLI still debated. Moreover, the observation of fatal pulmonary toxicity has limited the use of BU-MEL combined with WLI [12,19,25,26].

The Italian Sarcoma Group (ISG) and Scandinavian Sarcoma Group (SSG) designed treatment protocols to evaluate the impact of HDCT with BU-MEL in localized (ISG/SSGIII) and metastatic (ISG/SSG IV) ES patients [7,13]. For lung metastatic ES, the study showed that an intensive approach using HDCT and WLI at doses of 12 Gy for <14 years old and 15 Gy for >14 years old is feasible, and resulted in five-year OS probability 52% [13]. The subsequent protocol ISG/AIEOP EW2 (www.clinicaltrials.gov identifier: NCT02727387 accessed on 2 June 2019) was designed to evaluate the addition of six months maintenance treatment after the same combination BU-MEL and WLI [27].

The aim of this study was to assess toxicity and predictors of outcome in ES patients with pulmonary and/or pleural metastases (PPM) treated in Italian centers with BU-MEL and WLI as part of two prospective and consecutive treatment protocols.

## 2. Materials and Methods

### 2.1. Setting and Staging

This multicentric study collected data from ES patients with PPM at time of diagnosis treated with WLI after BU-MEL in eleven Italian oncology centers. Patient recruitment into trials was carried out from 1 November 1999 to 31 May 2017. The follow-up data are for 30 June 2019.

All patients were treated according to ISG/SSG-IV and AEIOP/ISG EW-2 protocol studies designed by ISG and AIEOP [13,27]. Study protocols were approved by an independent ethics committee and the institutional review boards. Written informed consent was obtained from all patients and/or their parents/guardians before enrollment.

All patients had biopsy-proven diagnosis of ES. Histological confirmation was obtained by a panel of expert pathologists and the diagnosis was molecularly assessed in 64 cases, while in 4 cases diagnosis of ES was confirmed by immunohistochemistry if CD99 marker staining resulted strong, diffuse and membranous and additional immunohistochemical testing excluded the diagnosis of lymphoblastic leukemias/lymphomas or other round and spindle cell sarcomas.

Patients were considered metastatic at lung if they had at least one pulmonary/pleural nodule >0.5 cm at chest CT scan.

### 2.2. Consolidation Treatment

In ISG/SSG-IV and AIEOP/ISG EW-2 protocols, consolidation treatment was performed with HDCT and WLI. HDCT combined BU-MEL followed by autologous hematopoietic stem cell transplantation (ASCT) of at least 2.5 × 10^6^ CD34+ cells/Kg of body weight. Oral busulfan at a dose of 1 mg/Kg or intravenous busulfan at a dose of 0.8 mg/Kg were administered every 6 h over 4 days, for a total of 16 doses. For pediatric patients with a weight <34 Kg intravenous busulfan doses changed according to drug data sheet. Melphalan was administered at a unique dose of 140 mg/m^2^ at least 24 h after the last dose of busulfan. In overweight patients, the method for determining the weight for chemotherapy calculation was adjusted ideal body weight [28].

WLI was delivered with a minimum interval of 8 weeks from ASCT. Total dose was 15 Gy in patients aged ≥14 years and 12 Gy in patients aged <14 years. Treatment was delivered in 10 fractions using three dimensional conformal technique (3D-CRT) or intensity modulated radiation therapy (IMRT) or volumetric modulated ARC therapy (VMAT). Bilateral whole lungs from the diaphragm to the apices represented the clinical target volume (CTV). The planning target volume (PTV) was defined as the CTV with an expansion of 0.5–1 cm in all directions. The dose distribution was prescribed at the isocenter based on ICRU reports. Patients were immobilized using a thermoplastic mask or individualized immobilization system.

Patients whose metastases did not respond to induction chemotherapy underwent metastasectomy whenever feasible before or after receiving WLI.

### 2.3. Toxicity Evaluation

To evaluate the impact of the different treatment modalities on the respiratory and cardiovascular function, each protocol design provided spirometry, echocardiogram and chest CT scan to be assessed during and after treatments. Spirometry and echocardiogram were evaluated before BU-MEL, before and after WLI and once a year during follow-up, unless otherwise indicated. Chest CT was repeated every 3 months in the first year, every 4 months during the second and third year, every 6 months during the fourth and fifth year then every year during follow-up. Endocrine disorders were performed once a year during follow-up, unless otherwise indicated. Toxicity was evaluated using CTCAE v. 4.03 [29].

Analysis of risk factors for the WLI-related toxicity included defined clinical features as gender, age (14 years or more versus <14 years), Body Mass Index (BMI), busulfan administration (intravenous or oral) and WLI technique applied. BMI was calculated according to standard formula [30]. Pediatric patients were classified as normal weight if the BMI was between 5° and 95° percentile, overweight if BMI was >95° percentile and underweight if the BMI was <5° percentile [31]. In order to assess the role of the overweight condition as a risk factor for any WLI-related complications, normal weight and underweight patients were considered together when compared to overweight patients.

### 2.4. Response and Outcome Evaluation

Response to induction chemotherapy of the primitive tumor site was evaluated by MRI. Histological tumor necrosis after neoadjuvant chemotherapy was evaluated according to the Bologna System [32] as following: Presence of macroscopic foci of viable tumor cells was graded I and the pathologic response was classified as poor; presence of isolated microscopic nodules of viable tumor cells (graded II) or absence of viable tumor (grade III) were both classified as pathologic good response. The pulmonary metastases response after induction chemotherapy was evaluated with chest CT according to RECIST criteria [33]. Pulmonary relapse was defined as recurrence in the lung, the pleural space or both. Timing and pattern of any site of relapse or progression from date of completion of WLI was analyzed.

The outcome analysis was per protocol, thus including only those patients enrolled in their respective protocols and who completed the scheduled treatment. Outcomes were overall survival (OS), event-free survival (EFS) and pulmonary relapse-free survival (PRFS). PRFS was chosen as an endpoint to analyze risk factors for pulmonary recurrence in those patients with only lung metastases who reached complete remission of lung lesions after induction chemotherapy. PRFS was defined as the time from the start of chemotherapy to pulmonary relapse or the last follow-up. Clinical variables analyzed as potential prognostic factor were: age <14 versus ≥14 years old, primary pelvic versus other tumor site, only lung metastases versus pulmonary and extra pulmonary metastases, complete (CR) versus partial response (PR) or stable disease (SD) of the soft tissue tumor component, surgery versus surgery plus radiotherapy versus definitive radiotherapy, good versus poor histological necrosis for patients who underwent surgery, number of lung metastases (≥10 vs <10), bilateral versus monolateral lung metastases, CR versus PR or SD of the lung metastases evaluated by CT, intravenous versus oral busulfan route of administration and time interval ≥90 days vs <90 days from ASCT to WLI start.

### 2.5. Data Collection and Statistical Analysis

Collected data were included in a new database, analyzed and evaluated by a statistician and two independent researchers, a pediatric oncologist and a radiation oncologist.

Demographic and clinical characteristics were summarized with descriptive statistics. Toxicity incidence with 95% confidence interval (CI) was calculated. Chi square test or Fisher exact test was used to evaluate the impact of risk factors for the WLI-related toxicity.

Outcomes were estimated by Kaplan–Meier methods and differences between groups were evaluated by log-rank test. A Cox proportional-hazards regression model was used to estimate adjusted hazard ratios (HRs) and 95% CIs. All variables statistically significant in univariate analysis were included in the regression model using the stepwise selection method. Statistical significance was set at *p* < 0.05. All statistical analyses were performed using SAS version 9.4.

## 3. Results

Data from sixty-eight lung metastatic ES patients treated with BU-MEL followed by WLI were collected. All patients resulted evaluable for the analysis. The clinical characteristics of the study population are listed in Table 1.

WLI started with a median time of 77 days from ASCT (range 56–178 days). Some patients did not undergo spirometry at the scheduled timing for reasons not reported. Pathologic spirometries were mainly restrictive and only grade 1 or 2. An increased incidence of pathologic spirometry was observed either after BU-MEL or after WLI (Table 2). The majority of patients were asymptomatic at last follow-up even with a pathologic spirometry.

Radiological toxicity was evaluated as pulmonary fibrosis, alveolitis or combination of both (Table 3). With a median time of 57 days after WLI, 26.5% of patients presented signs of radiological toxicity mainly grade 1 or grade 2. Pulmonary involvement showed to improve and resolve over time and in none of the patients with radiological toxicity symptoms were reported at the last follow-up.

Gender, age, administration route of busulfan and body mass index were analyzed as risk factors and no statistical significance for pathologic spirometry or radiological toxicity was found.

One or more clinical toxicities were reported in 15 (22.1%) patients. The most common clinical toxicity was pneumonitis (Table 4). In 11 (16.2%) patients clinical toxicities were grade 1 or grade 2. One or more grade 3 clinical toxicity were reported in 4 (5.9%) patients and in all cases developed within two months from WLI. All grade 3 toxicities resolved during the follow-up.

No cardiac dysfunction or significant change in echocardiographic parameters evaluated during treatment or follow-up was reported in these series. Finally, as regards late toxicities, hypothyroidism was reported in only one patient who needed thyroid hormone replacement.

**Figure 1:**
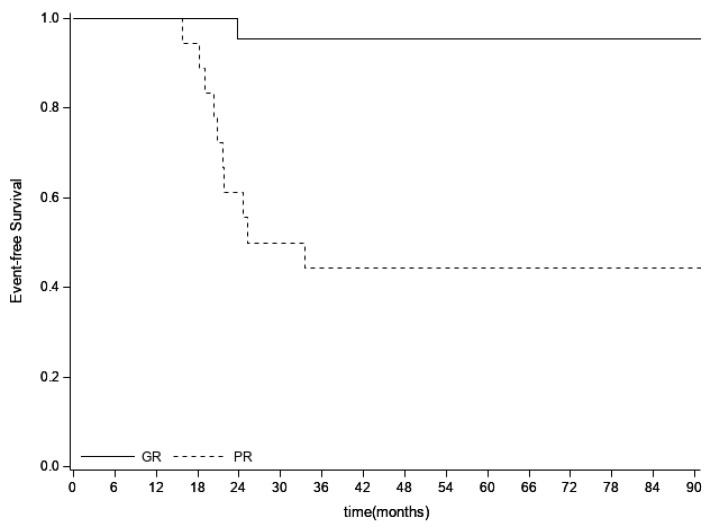
Event-free survival by primary tumor chemotherapy-induced histological necrosis in lung metastatic Ewing sarcoma patients. GR = histological good response, PR = histological poor response.

As of 30 June 2019, the median follow-up was 86 months (95%CI = 68–118). Twenty-six events occurred with a median time to progression from WLI of 10 months (range = two to 46 months). The three-year and five-year OS were 72.9% (95%CI = 60.4–82) and 69.8% (95%CI = 57.1–79.3), respectively, while the three-year and five-year EFS were 63.1% (95%CI = 50.4–73.3) and 61.2% (95%CI = 48.4–71.7), respectively. Univariate analysis for Event-Free Survival showed statistical significance for primary site (*p* = 0.0040), type of local therapy (*p* = 0.0005), histological necrosis of the primary tumor (*p* = 0.0003), chest CT chemotherapy response (*p* = 0.0218) and site of metastases (*p* = 0.0178) (Table 5). Multivariate analysis confirmed a decreased risk of disease relapse or progression for patients with good histological necrosis compared to patients with poor histological necrosis of the primary tumor [HR = 0.06 (95%CI = 0.01–0.47), *p* = 0.0073] and for patient who had surgery or surgery with radiotherapy as a local treatment compared to patients who had radiotherapy alone [HR = 0.18 (95%CI = 0.04–0.79), *p* = 0.0235]. Moreover, patients with good histological necrosis showed a significant decreased risk of death compared to patients with poor histological necrosis of the primary tumor [HR = 0.08 (95%CI = 0.01–0.63), *p* = 0.0163]. The results shown for EFS by primary tumor histological necrosis (Figure 1) were similar to those observed for OS.

The three-year and five-year PRFS resulted 78.1% (95%CI = 61–88.4) and 70.5% (CI = 56.3–80.8), respectively. Among these patients who had complete remission of lung lesions, no event occurred in the subgroup with good histological response of the primary tumor while those with poor histological response showed a higher risk of pulmonary relapse (Χ^2^ = 10.85, *p* = 0.0010).

## 4. Discussion

Few published data to evaluate the feasibility of WLI combined with high-dose chemotherapy are available, due to the relatively limited number of cohorts and the lack of treatment homogeneity across the studies [34]. This study is the largest report on toxicity and outcome following BU-MEL and WLI in lung metastatic ES.

The peculiarity of this study lies in the use of lower doses (12 or 15 Gy) than the standard doses recommended by the international guidelines [20]. The reason for the choice of lower doses lies on the higher risk of severe pulmonary toxicities expected with the combination of BU-MEL and standard dose WLI, as reported in other cooperative studies [12,25,26].

After the combined treatment BU-MEL and WLI, almost a quarter of patients developed radiological or clinical toxicity, in the majority of cases CTCAE grade 1 or 2. Only 5.9% of patients developed one or more grade 3 clinical toxicity, all resolved during the follow-up. Overall, toxicities observed were manageable and therefore we can consider the association of BU-MEL and WLI at reduced doses as feasible. However, our study demonstrated that a high level of monitoring patients is required both by radiological examination and functional respiratory tests, particularly in the first months after treatment.

The minimum time interval of eight weeks between BU-MEL and WLI may have contributed to limit the incidence and the grade of the observed toxicities. In a recent review, a time interval of 30–60 days between HDCT and WLI showed a significant impact on pulmonary function disorders [34].

Regarding late toxicities, we reported only one patient who developed grade 2 hypothyroidism requiring thyroid hormone replacement. Screening for thyroid function during follow-up, including thyroid ultrasound in selected cases, as well as a longer follow-up are needed to determine the onset of thyroid disorders and secondary thyroid malignancies in this cohort of patients.

The Euro-Ewing-Intergroup EE99 and Ewing 2008 trials, which randomized patients with pulmonary metastatic disease alone to HDCT with BU-MEL or to standard consolidation chemotherapy with WLI, reported no statistically significant difference between the two arms in terms of survival [14]. Since in these trials WLI was combined only to standard chemotherapy, the comparison of outcomes with our study considered the BU-MEL arm of the as-treated population, which included 123 patients, and the group of 56 patients in our series with only lung metastases. The three-year EFS of 57.7% (48.8–66.2) were poorer compared with the three-year EFS of 69.5% (55.6–79.8) of our study. Due to the different nature and treatment modality between the two studies, the benefit of adding WLI to HDCT with BU-MEL can only be assumed and should be confirmed in a randomized study.

Previous studies found complete resolution of lung metastases after induction chemotherapy to correlate with survival in ES [13,18,34]. Our data confirm this observation. However, the strongest correlation was observed between chemotherapy-induced histological necrosis of the primary tumor and survival. In particular, by analyzing the three-year and five-year PRFS, pulmonary relapses occurred only in patients with poor histological response of the primary tumor. Since these are all patients with complete response of the lung lesions after induction chemotherapy, it could be speculated that in some histologically poor-responder patients the combined strategy BU-MEL with WLI was not able to eradicate the microscopic malignant cells in the lung. These results raise the question of whether a different treatment stratification based on the histological response of the primary tumor is needed in lung metastatic ES.

As a future direction, less toxic RT or high-dose chemotherapy regimens should be explored too. Modern RT techniques, including intensity-modulated radiotherapy (IMRT), proton therapy, superior cardiac protection and 4D lung volumes dose coverage, might reduce the risk of treatment-related adverse effects [23,35]. High-dose chemotherapy regimens containing treosulfan, which is a hydrophilic analogue of busulfan, should be more extensively evaluated in combination with WLI [24], since treosulfan is expected to have a better toxicity profile than busulfan [36].

## 5. Limitations

The main limitation of this study was its retrospective nature, although all patients were enrolled into the two prospective and consecutive ISG/AIEOP treatment protocols for metastatic ES. The long study period is also to be considered. However, the eighteen-years study period was chosen because the consolidation phase of the two consecutive treatment protocols remained substantially unchanged over time, allowing us to assess even very-late toxicities. Some patients did not undergo spirometry at the scheduled timing and this may have partially influenced the assessment of pulmonary toxicity after WLI. Furthermore, the outcome analysis was per protocol, since the participating Centers were asked to send only data of those patients who completed the scheduled treatment. This is the main reason of better survival curves observed for this study compared to the intention to treat analysis previously reported [13].

## 6. Conclusions

Our data suggest that WLI at recommended doses and time intervals after BU-MEL is feasible and might contribute to reduce the pulmonary relapse and the risk of death in lung metastatic ES with responsive disease. Differently, further studies are needed to better define the treatment strategies in the group of patients with poor histological response of the primary tumor. Finally, the results of this study provide support for considering the treatment stratification based on the histological response of the primary tumor in ES patients with lung metastases.

Looking forward to innovative therapies, a prospective randomized trial should be undertaken to compare the different treatment strategies in terms of efficacy and toxicity.

## Figures and Tables

**Table 1:** Patients characteristics.

Characteristics		N	%
Median age (range)	14 years (8 months–35 years)			
Gender	Male	39	57.3
	Female	29	42.7
Trial	ISG/AIEOP EW2	36	52.9
	ISG/SSG IV	32	47.1
Primary tumor site	Extremities	33	48.5
	Pelvis	21	30.9
	Vertebrae	4	5.9
	Ribs	5	7.3
	Other	5	7.3
Metastases	Only lung	56	82.3
	Lung + extra pulmonary	12	17.7
	Lung bilateral	52	76.5
	Lung monolateral	16	23.2
Local treatment	Surgery ^1^	33	48.5
	Surgery + radiotherapy ^2^	16	23.5
	Radiotherapy	19	27.9
Metastasectomy	Lung	4	5.9
	Bone	1	1.5
Busulfan administration	IV	42	61.8
	Oral	26	38.1
Radiotherapy technique	3D	50	73.5
	IMRT/VMAT	18	26.5

^1^ One patient had surgery after busulfan/melphalan (BU-MEL); ^2^ four patients had surgery after radiotherapy and BU-MEL.

**Table 2:** Pathologic spirometry–incidence and grade.

Spirometry	Pre-BuMel	Pre-WLI	Post-WLI	Last Follow-Up
No. of patients	61	38	55	44
Restrictive	4	9	17	15
Grade 1	3	7	13	10
Grade 2	1	2	4	5
Grade 3	-	-	-	-
Obstructive	1	0	3	1
Grade 1	1	-	3	-
Grade 2	-	-	-	1
Grade 3	-	-	-	-
No. (%) pathologic	5 (8.2%)	9 (23.7%)	20 (36.4%)	16 (36.4%)
95% CI	3.4–18.2%	12.8–39.6%	24.8–49.7%	23.6–51.4%

**Table 3:** Radiological toxicity—incidence and grade in 68 patients.

Toxicity CTCAE Grade	Pts	%	95% C.I.
**Pulmonary Fibrosis** Grade 1 2	7 3 4	10.3	5–20.1%
**Alveolitis**	4	5.9	2.2–14.6%
Grade 1 2 3	2 1 1		
**Pulmonary Fibrosis + Alveolitis** Grade 1 2	7 5 2	10.3	5–20.1% 17.4–38.2%
**TOTAL** Grade 1 2 3	18 10 7 1	26.5	

**Table 4:** Clinical toxicity—incidence and grade in 68 patients.

Toxicity CTCAE Grade	Pts	%	95% C.I.
**Pneumonitis/Pneumonia** Grade 1 2	6 2 4	8.8	4–18%
**Cough** Grade 1 2	5 1 4	7.4	3.1–16.3%
**Esophagitis** Grade 1 2	4 1 3	5.9	2.2–14.6%
**Anorexia** Grade 2	3 3	4.3	1.4–12.6%
**Asthenia** Grade 2	3 3	4.3	1.4–12.6%
**Nausea** Grade 2	3 3	4.3	1.4–12.6%
**Odynophagia** Grade 2 3	3 2 1	4.3	1.4–12.6%
**Dyspnea** Grade 1	1 1	1.5	0.2–9.6%
**Hypothyroidism** Grade 2	1 1	1.5	0.2–9.6%

Some patients experienced more than one toxicity.

**Table 5:** Univariate analysis for event-free survival.

Variable		Pts	Events 3y	%EFS 3y (95%CI)	Events 5y	%EFS 5y (95%CI)	*p* Value
**Age**	≤14 y	31	12	61 (41.6–75.7)	12	61 (41.6–75.7)	0.9634
	>14 y	37	13	64.9 (47.3–77.9)	14	61.6 (43.9–75.2)	
**Primary site**	Pelvic	21	12	42.9 (21.9–62.3)	13	38.1 (18.3–57.8)	0.0040
	Other	47	13	72.1 (56.8–82.8)	13	72.1 (56.8–82.8)	
**Soft tissue response**	CR	18	5	71.1 (43.8–86.9)	5	71.1 (43.8–86.9)	0.2295
	PR/SD	49	20	59.2 (44.2–71.4)	21	56.7 (41.6–69.3)	
**Local therapy**	Surgery	33	9	72.4 (53.6–84.6)	9	72.4 (53.6–84.6)	0.0005
	Surgery + RT	16	3	81.3 (52.5–93.5)	4	74.5 (45.4–89.6)	
	RT	19	13	31.6 (12.9–52.2)	13	31.6 (12.9–52.2)	
**Histological necrosis**	Good	22	1	95.5 (71.9–99.3)	1	95.5 (71.9–99.3)	0.0003
	Poor	18	10	44.4 (21.6–65.1)	10	44.4 (21.6–65.1)	
**Metastatic site**	Only lung	56	17	69.5 (55.6–79.8)	18	67.2 (53–78)	0.0178
	Lung + extra pulmonary	12	8	33.3 (10.3–58.8)	8	33.3 (10.3–58.8)	
**Lung** **metastases**	≤10	46	14	69.5 (54–80.7)	15	66.6 (50.7–78.4)	0.2485
	>10	22	11	50 (28.2–68.4)	11	50 (28.2–68.4)	
	Bilateral	52	21	59.3 (44.7–71.3)	21	59.3 (44.7–71.3)	0.5647
	Monolateral	16	4	75 (46.3–71.3)	5	65.6 (34.9–84.5)	
**Chest CT response**	CR	42	11	73.8 (57.7–84.6)	12	70.7 (54.1–82.3)	0.0218
	PR/SD	26	14	45.6 (25.9–63.3)	14	45.6 (25.9–63.3)	
**Busulfan**	IV	42	19	54.5 (38.3–68.1)	19	54.5 (38.3–68.1)	0.0824
	OS	26	6	76.9 (55.7–88.9)	7	72.9 (51.4–86.1)	
**WLI start from ASCT**	>90 days	17	7	58.8 (32.5–77.8)	7	58.8 (32.5–77.8)	0.8484
	≤90 days	51	18	64.5 (49.8–76)	19	61.8 (46.8–73.8)	

CR = complete response, PR = partial response, SD = stable disease, RT = radiotherapy, IV = intravenous, OS = oral.

## References

[1] Manfrini M., Fabbri N., Picci P., Gambarotti M., Vanel D. (2014). Atlas of Musculoskeletal Tumors and Tumorlike Lesions.

[2] Wolden S.L., Alektiar K.M. (2010). Sarcomas across the Age Spectrum. Semin. Radiat. Oncol..

[3] Bölling T., Schuck A., Paulussen M., Dirksen U., Ranft A., Könemann S., Dunst J., Willich N., Jürgens H. (2008). Whole Lung Irradiation in Patients with Exclusively Pulmonary Metastases of Ewing Tumors. Strahlenther Onkol..

[4] Grier H.E., Krailo M.D., Tarbell N.J., Link M.P., Fryer C.J.H., Pritchard D.J., Gebhardt M.C., Dickman P.S., Perlman E.J., Meyers P.A. (2003). Addition of Ifosfamide and Etoposide to Standard Chemotherapy for Ewing’s Sarcoma and Primitive Neuroectodermal Tumor of Bone. N. Engl. J. Med..

[5] Paulussen M., Craft A.W., Lewis I., Hackshaw A., Douglas C., Dunst J., Shuck A., Winkelmann W., Köhler G., Poremba C. (2008). Results of the EICESS-92 Study: Two Randomized Trials of Ewing’s Sarcoma Treatment—Cyclophosphamide Compared With Ifosfamide in Standard-Risk Patients and Assessment of Benefit of Etoposide Added to Standard Treatment in High-Risk Patients. J. Clin. Oncol..

[6] Granowetter L., Womer R., Devidas M., Krailo M., Wang C., Bernstein M., Marina N., Leavey P., Gebhardt M., Healey J. (2009). Dose-Intensified Compared With Standard Chemotherapy for Nonmetastatic Ewing Sarcoma Family of Tumors: A Children’s Oncology Group Study. J. Clin. Oncol..

[7] Ferrari S., Sundby Hall K., Luksch R., Tienghi A., Wiebe T., Fagioli F., Alvegard T.A., Brach Del Prever A., Tamburini A., Alberghini M. (2011). Nonmetastatic Ewing family tumors: High-dose chemotherapy with stem cell rescue in poor responder patients. Results of the Italian Sarcoma Group/Scandinavian Sarcoma Group III protocol. Ann. Oncol..

[8] Womer R.B., West D.C., Krailo M.D., Dickman P.S., Pawel B.R., Grier H.E., Marcus K., Sailer S., Healey J.H., Dormans J.P. (2012). Randomized Controlled Trial of Interval-Compressed Chemotherapy for the Treatment of Localized Ewing Sarcoma: A Report from the Children’s Oncology Group. J. Clin. Oncol..

[9] Le Deley M.-C., Paulussen M., Lewis I., Brennan B., Ranft A., Whelan J., Le Teuff G., Michin J., Ladenstein R., Marec-Bérard P. (2014). Cyclophosphamide compared with ifosfamide in consolidation treatment of standard-risk Ewing sarcoma: Results of the randomized noninferiority Euro-EWING99-R1 trial. J. Clin. Oncol..

[10] Whelan J., Le Deley M.-C., Dirksen U., Le Teuff G., Brennan B., Gaspar N., Hawkins D.S., Amler S., Bauer S., Bielack S. (2018). High-Dose Chemotherapy and Blood Autologous Stem-Cell Rescue Compared With Standard Chemotherapy in Localized High-Risk Ewing Sarcoma: Results of Euro-E.W.I.N.G.99 and Ewing-2008. J. Clin. Oncol..

[11] Ladenstein R., Pötschger U., Le Deley M.C., Whelan J., Paulussen M., Oberlin O., van den Berg H., Dirksen U., Hjorth L., Michon J. (2010). Primary Disseminated Multifocal Ewing Sarcoma: Results of the Euro-EWING 99 Trial. J. Clin. Oncol..

[12] Oberlin O., Rey A., Desfachelles A.S., Philip T., Plantaz D., Schmitt C., Plouvier E., Lejars O., Rubie H., Terrier P. (2006). Impact of High-Dose Busulfan Plus Melphalan As Consolidation in Metastatic Ewing Tumors: A Study by the Société Française des Cancers de l’Enfant. J. Clin. Oncol..

[13] Luksch R., Tienghi A., Hall K.S., Fagioli F., Picci P., Barbieri E., Gandola L., Eriksson M., Ruggieri P., Daolio P. (2012). Primary metastatic Ewing’s family tumors: Results of the Italian Sarcoma Group and Scandinavian Sarcoma Group ISG/SSG IV Study including myeloablative chemotherapy and total-lung irradiation. Ann. Oncol..

[14] Dirksen U., Brennan B., Le Deley M.-C., Cozic N., van den Berg H., Bhadri V., Brichard B., Claude L., Craft A., Amler S. (2019). High-Dose Chemotherapy Compared With Standard Chemotherapy and Lung Radiation in Ewing Sarcoma With Pulmonary Metastases: Results of the European Ewing Tumour Working Initiative of National Groups, 99 Trial and EWING 2008. J. Clin. Oncol..

[15] Bacci G., Ferrari S., Longhi A., Donati D., De Paolis M., Forni C., Versari M., Setola E., Briccoli A., Barbieri E. (2003). Therapy and survival after recurrence of Ewing’s tumors: The Rizzoli experience in 195 patients treated with adjuvant and neoadjuvant chemotherapy from 1979 to 1997. Ann. Oncol..

[16] McTiernan A., Driver D., Michelagnoli M.P., Kilby A.M., Whelan J.S. (2006). High dose chemotherapy with bone marrow or peripheral stem cell rescue is an effective treatment option for patients with relapsed or progressive Ewing’s sarcoma family of tumours. Ann. Oncol..

[17] Ferrari S., Luksch R., Hall K.S., Fagioli F., Prete A., Tamburini A., Tienghi A., DiGirolamo S., Paioli A., Abate M.E. (2015). Post-relapse survival in patients with Ewing sarcoma: High-Dose Therapy in Recurrent Ewing Sarcoma. Pediatr. Blood Cancer.

[18] Dunst J., Paulussen M., Jürgens H. (1993). Lung irradiation for Ewing’s sarcoma with pulmonary metastases at diagnosis: Results of the CESS-studies. Strahlenther Onkol..

[19] Paulussen M., Ahrens S., Burdach S., Craft A., Dockhorn-Dworniczak B., Dunst J., Fröhlich B., Winkelmann W., Zoubek A., Jürgens H. (1998). Primary metastatic (stage IV) Ewing tumor: Survival analysis of 171 patients from the EICESS studies. Ann. Oncol..

[20] Biermann J.S., Chow W., Reed D.R., Lucas D., Adkins D.R., Agulnik M., Benjamin R.S., Brigman B., Budd G.T., Curry W.T. (2017). NCCN Guidelines Insights: Bone Cancer, Version 2.2017. J. Natl. Compr. Canc. Netw..

[21] Razek A., Perez C.A., Tefft M., Nesbit M., Vietti T., Burgert E.O., Kissane J., Pritchard D.J., Gehan E.A. (1980). Intergroup Ewing’s Sarcoma Study: Local control related to radiation dose, volume, and site of primary lesion in Ewing’s sarcoma. Cancer.

[22] Whelan J.S., Burcombe R.J., Janinis J., Baldelli A.M., Cassoni A.M. (2002). A systematic review of the role of pulmonary irradiation in the management of primary bone tumours. Ann. Oncol..

[23] Ronchi L., Buwenge M., Cortesi A., Ammendolia I., Frakulli R., Abate M.E., Arcelli A., Donati C.M., Macchia G., Morganti A.G. (2018). Whole Lung Irradiation in Patients with Osteosarcoma and Ewing Sarcoma. Anticancer Res..

[24] Scobioala S., Ranft A., Wolters H., Jabar S., Paulussen M., Timmermann B., Jürgens H., Hassenpflug W., Klingebiel T., Elsayad K. (2018). Impact of Whole Lung Irradiation on Survival Outcome in Patients With Lung Relapsed Ewing Sarcoma. Int. J. Radiat. Oncol. Biol. Phys..

[25] Ladenstein R., Lasset C., Pinkerton R., Zucker J.M., Peters C., Burdach S., Pardo N., Dallorso S., Coze C. (1995). Impact of megatherapy in children with high-risk Ewing’s tumours in complete remission: A report from the EBMT Solid Tumour Registry. Bone Marrow Transpl..

[26] Spunt S.L., McCarville M.B., Kun L.E., Poquette C.A., Cain A.M., Brandao L., Pappo A.S. (2001). Selective Use of Whole-Lung Irradiation for Patients with Ewing Sarcoma Family Tumors and Pulmonary Metastases at the Time of Diagnosis. J. Pediatr. Hematol. Oncol..

[27] Puma N., Asaftei D.S., Paioli A., Bisogno G., Rabusin M., Coccoli L., Tamburini A., Milano G.M., Mascarin M., Bertulli R. (2020). Maintenance therapy with oral cyclophosphamide plus celecoxib in patients with metastatic Ewing sarcoma: Results of the Italian Sarcoma Group/AIEOP EW-2 study. J. Clin. Oncol..

[28] Bauer L.A., Edwards W.A.D., Dellinger E.P., Simonowitz D.A. (1983). Influence of weight on aminoglycoside pharmacokinetics in normal weight and morbidly obese patients. Eur. J. Clin. Pharmacol..

[29] Basch E., Bennett A., Pietanza M.C. (2011). Use of patient-reported outcomes to improve the predictive accuracy of clinician-reported adverse events. J. Natl Cancer Inst..

[30] Mei Z., Grummer-Strawn L.M., Pietrobelli A., Goulding A., Goran M.I., Dietz W.H. (2002). Validity of body mass index compared with other body-composition screening indexes for the assessment of body fatness in children and adolescents. Am. J. Clin. Nutr..

[31] Kuczmarski R.J., Ogden C.L., Grummer-Strawn L.M., Flegal K.M., Guo S.S., Wei R., Mei Z., Curtin L.R., Roche A.F., Johnson C.L. (2000). *CDC growth charts*: United States. Adv. Data.

[32] Picci P., Böhling T., Bacci G., Ferrari S., Sangiorgi L., Mercuri M., Ruggieri P., Manfrini M., Ferraro A., Casadei R. (1997). Chemotherapy-induced tumor necrosis as a prognostic factor in localized Ewing’s sarcoma of the extremities. J. Clin. Oncol..

[33] Eisenhauer E.A., Therasse P., Bogaerts J., Schwartz L.H., Sargent D., Ford R., Dancey J., Arbuck S., Gwyther S., Mooney M. (2009). New response evaluation criteria in solid tumours: Revised RECIST guideline (version 1.1). Eur. J. Cancer.

[34] Scobioala S., Eich H.T. (2020). Risk stratification of pulmonary toxicities in the combination of whole lung irradiation and high-dose chemotherapy for Ewing sarcoma patients with lung metastases: A review. Strahlenther Onkol..

[35] Mazzola R., Ruggieri R., Figlia V., Rigo M., Giaj Levra N., Ricchetti F., Nicosia L., Corradini S., Alongi F. (2019). Stereotactic body radiotherapy of central lung malignancies using a simultaneous integrated protection approach. Strahlenther Onkol..

[36] Rasper M., Jabar S., Ranft A., Jürgens H., Amler S., Dirksen U. (2014). The value of high-dose chemotherapy in patients with first relapsed Ewing sarcoma. Pediatr. Blood Cancer.

